# Effect of ultrasound-guided fascia iliaca compartment block on pain management in hip fracture patients: A double-blind placebo-controlled randomized clinical trial

**DOI:** 10.1097/MD.0000000000044622

**Published:** 2025-10-03

**Authors:** Rory D. O’Connor, Sanne Postma, Djamila Van Kamp, Annelies C.E. Benraad, Alicia Lommen, Sarah Körver, Jan A. Ten Bosch, Mattheus K. Reinders

**Affiliations:** a Emergency Department, Henri Dunantstraat 1, 's‐Hertogenbosch, The Netherlands; b Emergency Department, Zuyderland Medical Centre, Heerlen, The Netherlands; c Department of Trauma Surgery, Maastricht University Medical Center, Maastricht, The Netherlands.

**Keywords:** analgesia, emergency service, hip fractures, levobupivacaine, nerve block, opioid analgesics, pain measurement, patient-controlled, randomized controlled trials as topic

## Abstract

**Background:**

Evidence for emergency department (ED) ultrasound-guided fascia iliaca compartment block (UG-FICB) in hip-fracture analgesia is limited.

**Methods:**

In this double-blind, placebo-controlled, randomized clinical trial in a Dutch ED, adults with a radiologically confirmed hip fracture were randomized 1:1 to weight-adjusted levobupivacaine UG-FICB or volume matched saline. All participants were granted access to a patient-controlled analgesia (PCA) pump with morphine for pain control. The primary outcome was cumulative morphine demand during the first 6 hours. Secondary outcomes were pain scores, time to first morphine demand, opioid-free period, and adverse events.

**Results:**

Fifty-five participants were randomized (January 28, 2019–February 17, 2020; 2 were excluded because surgery occurred within an hour after UG-FICB, leaving 53 for analysis (levobupivacaine 29, placebo 24). Median 6 hours morphine demand was 3 mg (1–6 with levobupivacaine and 3 mg (2–6) with placebo (*P* = .46). Time to first morphine request was shorter on placebo (HR 1.87, 95% confidence interval (CI): 1.01–3.45) and levobupivacaine increased the opioid-free proportion (24% vs 4). Pain at 1 hour was lower with levobupivacaine (mean difference was –1.5 NRS units, *P* = .02); thereafter differences were non-significant. The following adverse events occurred: nausea/vomiting 3, injection pain 1, delirium 1, drowsiness 1). Recruitment stopped early because of COVID-19 pandemic.

**Conclusions:**

Single-shot UG-FICB did not reduce 6 hours morphine consumption, but delayed first opioid use and allowed one quarter of patients to remain opioid-free. Larger adequately powered trials are required.

## 1. Introduction

Hip fractures are common presentations in the emergency department (ED). In the Netherlands, the nationwide incidence of hip fractures were 120 per 100,000 person-years in 2019, with rates climbing to 548 per 100,000 among those aged over 65 years.^[1]^ The resulting care costs the Dutch health system estimated at €425 million per year with one-year mortality estimated around 20%.^[2]^ A major challenge in hip fracture patients is pain management. Suboptimal pain control is associated with stress, a prolonged hospital stay and delirium.^[3]^ Pain or fear of pain can limit early mobilization, an important goal in treatment. Therefore, early pain management is essential. Standard analgesic care consists of acetaminophen, opiates and/or non-steroidal anti-inflammatory drugs (NSAID’s). NSAIDs are contra-indicated in many older adults because of gastrointestinal bleeding and renal impairment.^[4]^ Opiates can cause respiratory depression, constipation, drowsiness, nausea and vomiting. Especially elderly patients are at risk for these side effects.^[5–7]^ The fascia iliaca compartment block (FICB) is widely used to treat patients with hip fractures, primarily due to its ease of application and favorable safety profile.^[8]^ Emergency physicians (EPs) frequently perform this block, generally under ultrasound guidance (UG-FICB), which enhances procedural safety and success rates.^[9,10]^

Nonetheless, UG-FICB takes more time and resources than systemic analgesia.^[11,12]^ Given the high ED workload, staffing constraints and time pressures, EPs need robust, high-quality evidence of clear benefits over simpler systemic analgesia before adopting UG-FICB as routine practice.^[13]^

Current evidence regarding the effectiveness of FICB remains limited and inconsistent. Systematic reviews by Steenberg et al and Fadhlillah et al demonstrated that FICB provides no statistically significant analgesic benefit over systemic analgesia at rest.^[14,15]^ In contrast, Wan et al reported that FICB might be superior to systemic analgesia, yet acknowledged considerable heterogeneity and controversy within included studies.^[16]^

Previous randomized controlled trials (RCTs) have mainly focused on pain during movement, which, although favoring FICB, carries limited clinical relevance as hip fracture patients are often immobilized preoperatively.^[15,17–19]^

Furthermore, earlier studies generally suffer from methodological limitations, such as high risk of bias, absence of ultrasound guidance (now widely considered standard care), lack of EP involvement, and reliance on nurse-administered opiate analgesia rather than patient-controlled analgesia (PCA), which better reflects individual pain experience and analgesic demand.^[15,17–19]^

A recent high-quality RCT by Pasquier et al involving EPs did not find significant analgesic benefits of FICB over placebo.^[18]^ However, ultrasound guidance was notably absent in their methodology. Thus, significant uncertainty persists regarding whether EP-performed UG-FICB provides clinically meaningful analgesic benefits. This double-blinded, placebo controlled randomized clinical trial was specifically designed to address these shortcomings. We hypothesized that patients receiving levobupivacaine UG-FICB would require less morphine in the first 6 hours than those receiving placebo.

## 2. Methods

### 2.1. Study design

Participants in this double-blind, placebo controlled-RCT (registered on ClinicalTrials.gov with registration number NCT03846102) were enrolled from a Dutch ED, which is part of Zuyderland Medical Centre in Heerlen, The Netherlands. This ED experiences an average annual attendance rate of about 35,000 patients. The trial protocol received approval from the local medical ethical review board (approval number: 16-T-215) and adhered to the Declaration of Helsinki.^[20]^

Monitoring of the trial occurred after the initial 5 inclusions and subsequently upon its premature termination due to the coronavirus disease 2019 (COVID-19) pandemic.

### 2.2. Selection of participants

Recruitment began on January 28, 2019, and concluded prematurely due to the COVID-19 pandemic, with the last participant being enrolled February, 2020.

Adult patients were eligible for enrollment by the attending physician if they had a radiologically confirmed hip fracture, such as a femoral collum, pertrochanteric or periprosthetic fracture. If they did not meet any exclusion criteria (Table 1), the physician obtained written informed consent from the patient to participate in the trial and undergo the UG-FICB.

**Table 1:** Eligibility exclusions applied before randomization.

• Skin infection at injection site(s) • Neurological deficit of fractured leg • Risk of compartment syndrome of ipsilateral lower leg • Transfer to another hospital • Hip fracture with other definitive treatment than operation • Operation within an hour after admission • Multiple fractures (more than 1) or other distracting pain • Currently using opiates • Allergy to trial medication (morphine or levobupivacaine) • History of cognitive impairment • Inability to understand and quantify pain on the NRS • Inability to use a PCA-pump • Pregnancy • No ED physician or ED nurse available for procedure. • INR > 4 or similar high risk of bleeding

Patients meeting any of the criteria listed were not enrolled in the trial.

INR = international normalized ratio, NRS = numerical rating scale, PCA = Patient-controlled analgesia, (0 = no pain, 10 = worst pain imaginable).

### 2.3. Interventions and data collection

For the UG-FICB, blinded syringes containing either levobupivacaine (treatment group) or normal saline (placebo group) were utilized. The syringes were prepared by the hospital pharmacy according to a randomization list, which was generated using an independent online randomization tool.^[21]^ Participants were randomly allocated in a 1:1 ratio to either the intervention or placebo group using block randomization (block size of 6). This randomization list and participant allocation remained concealed until the RCT concluded with the last participant. However, participant allocation could be disclosed in case of emergencies such as local anaesthetic systemic toxicity reactions.

As levobupivacaine is dosed based on ideal body weight (IBW), participants received their appropriate dose of blinded trial medication. The hospital pharmacy approximated this dose by assembling sets of 3 syringes (A, B and C) containing the same blinded substance (levobupivacaine or sodium chloride) at 3 different dosages and corresponding volumes, tailored to predetermined IBW categories (Table 2).

**Table 2:** Weight-based dosing scheme for trial medication.

Syringe contents	Weight categories (IBW)		
	≤65 kg	65–75 kg	≥75 kg
Treatment group: levobupivacaine			
Levobupivacaine (mg)	100	130	150
Volume (mL)	40	45	50
Placebo group: sodium chloride			
Volume (mL)	40	45	50

Syringe contents were adjusted to ideal-body-weight (IBW) bands: ≤65 kg, 65–75 kg, and ≥75 kg. Patients in the treatment arm received levobupivacaine 100, 130, or 150 mg (total volumes 40, 45, and 50 mL, respectively). Matching volumes of 0.9% sodium-chloride placebo were prepared for control patients.

IBW = ideal body weight.

After obtaining informed consent, an EP or emergency resident performed the UG-FICB as described by Haines et al.^[22]^ Interobserver variability for the UG-FICB procedure was not explicitly assessed. To minimize variability, all physicians involved received standardized hands-on training and had each performed a minimum of 10 successful UG-FICB prior to patient enrollment.

The EP selected one of the 3 syringes that matched the participant’s IBW category. The remaining 2 syringes from the set were returned to the pharmacy along with the sealed safety envelope. The pharmacy ensured an adequate stock of sets within the ED at all times.

After the UG-FICB procedure, an ED nurse confirmed the presence of an intravenous canula and connected it to a PCA pump. The PCA pump was loaded with a syringe containing a 1 mg/ml morphine solution and was pre-programmed with a 1 ml bolus and 6-minute lockout time. The EP or a nurse provided the participant with instructions how to use the PCA pump.

Additionally, each participant was provided with a tablet computer equipped with a self-developed pain-scoring application. While awaiting hip fracture surgery, participants were admitted to a hospital ward.

The trial’s conclusion was marked either by the participant proceeding for surgery or the passage of 24 hours since the UG-FICB administration.

Recorded participant characteristics encompassed age, gender, American Society of Anesthesiologists score, weight in kilograms, height in meters and the type of hip fracture. Moreover, the analgesia administered to participants by paramedics before their arrival at the ED was also recorded.

Furthermore, nurses asked participants to assess their pain using a 10-point scale, commonly known as the numerical rating scale (NRS). The NRS is a validated pain-assessment tool ranging from 0 (no pain) to 10 (worst imaginable pain).^[23]^ In the ED, baseline scores were collected immediately before UG-FICB administration. In the ward, participants recorded hourly scores via a tablet-based application, supplemented by nurse assessments at regular shift changes, ensuring comprehensive pain monitoring.

As it was imperative to access the internal data of the PCA pump, a specific model (Agilia SP PCA WiFi) was provided by Fresenius Kabi Netherlands b.v. for the trial. The data of this model could be easily retrieved by downloading onto a computer from the internal memory. This dataset contained records of each instance when a participant attempted self-dosing with the PCA pump. Using these recorded time points, the cumulative use of morphine was calculated in milligrams for each hour.

### 2.4. Outcome measures

The primary outcome was cumulative morphine demand during the first 6 hours following UG-FICB administration, measured using the internal memory of the PCA pump. The secondary outcomes were:

1. Pain scores measured using the NRS, recorded at baseline and then hourly a both tablet-based application, and additionally by nursing staff 3 times daily.
2. The proportion of participants who refrained from using any additional morphine.
3. Time to first morphine request, recorded from the PCA pump log.
4. Incidence of adverse events following the UG-FICB procedure.

All outcomes were assessed from the time of block administration until surgery or 24 hours post-intervention, whichever occurred first.

### 2.5. Data analysis

The trial’s sample size was determined to be 120 participants, based on an effect size of 0.67 mg per hour,^[24]^ an alpha (α) of 0.05 and a statistical power of 95%.

Data distribution was assessed using the Shapiro–Wilk test and visual methods. Age, weight, height, body-mass index, and pain scores were presented as mean ± standard deviation (SD). Time to operation, trial time, time to first morphine request and morphine cumulative demand at 6 hours were reported as median with interquartile range (IQR). Categorical variables were reported as frequencies and percentages. For normally distributed variables, the Student *t* test was applied, while the Mann–Whitney *U* test was employed for non-parametric continuous data. Pearson’s chi-squared test was utilized to compare categorical variables.

The primary outcome, morphine demand in the first 6 hours, was analyzed with the Mann–Whitney *U* test. Because some participants proceeded to surgery before reaching this time point, a linear mixed model was employed to explore the relationship between UG-FICB and morphine demand, treating UG-FICB as the fixed effect and time as the random effect. A similar approach was applied to the secondary outcome pain scores.

Time to first morphine request was analyzed using Kaplan–Meier survival curves and compared between groups using the log-rank test. Additionally, a Cox proportional hazards regression model was used to estimate the hazard ratio and 95% confidence interval (CI) for morphine request in the placebo group relative to the levobupivacaine group.

An exploratory post hoc power calculation was undertaken for the primary outcome after database lock and reported as hypothesis generating.

Sample size calculations were performed using G*Power version 3.1.9.2 (Heinrich Heine University Düsseldorf, Germany) and GLIMMPSE (version 2.0.0).^[25]^ All statistical analyses were performed using IBM SPSS Statistics version 28.0.1.0 (IBM Corp., Armonk, NY).

## 3. Results

**Figure 1.**
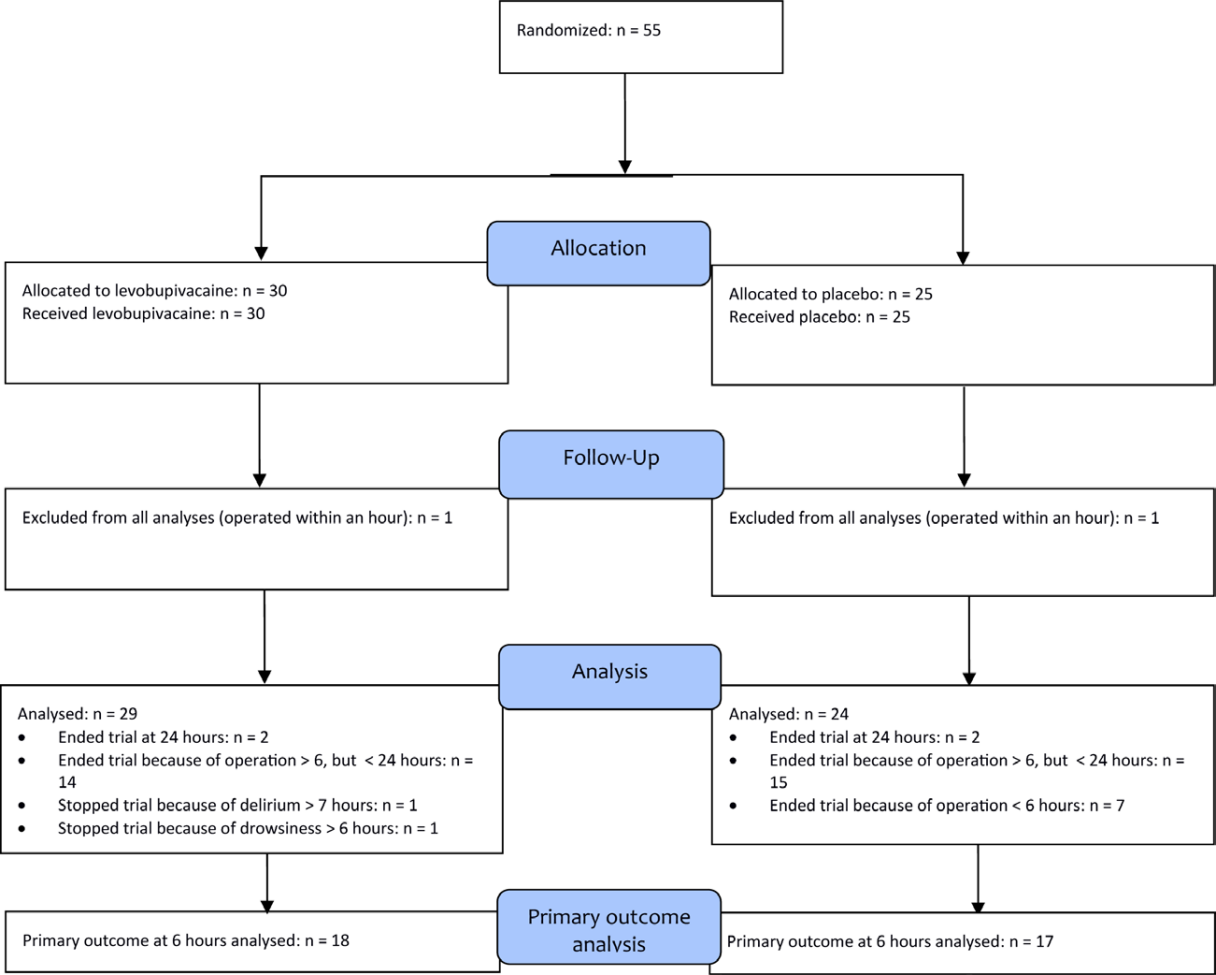
CONSORT flow diagram of participant allocation, follow-up and analysis. Fifty-five hip-fracture patients were randomized in the ED to a single-shot ultrasound-guided fascia iliaca block with levobupivacaine (n = 30) or saline placebo (n = 25). One participant in each arm proceeded to surgery within 1 h of block placement and was therefore excluded from analyses. Of the remaining 53 participants, in 35 (levobupivacaine n = 18; placebo n = 17) the primary 6-h opioid-consumption was assessed. Follow-up to 24 h was completed in 4 participants (2 per arm). Reasons for discontinuation before 24 h included earlier surgery (levobupivacaine n = 25; placebo n = 22), delirium (n = 1) and drowsiness (n = 1) in the intervention group. All participants were analyzed in the groups to which they were randomized. ED = emergency deparment.

For this trial 55 participants were enrolled, with 30 allocated to the intervention group and 25 to the placebo group (Fig. 1). Among each group, one participant proceeded for hip surgery within one hour after the UG-FICB, leading to their exclusion from all analyses. The remaining 53 participants were included in the analysis. The primary outcome at the 6-hour mark was evaluated in 35 participants. Other participants did not reach the predetermined 6-hour period as their hip surgeries were conducted earlier.

Baseline pain scores were similar between both groups before the UG-FICB (Table 3). A slightly number of participants in the placebo group had received analgesia before the procedure, from sources such as general practitioners, paramedics or ED staff, although this difference was not statistically significant.

**Table 3:** Baseline characteristics of trial participants.

	Levobupivacain (n = 29)			Placebo (n = 24)			*P*
	Mean	SD	n (%)	Mean	SD	n (%)	
Age (yr)	77	12		76	11		.79
Gender							
Female			15 (52)			9 (38)	.30
Male			14 (48)			15 (63)	
Weight (kg)	75	14		68	14		.08
Height (cm)	172	9		167	9		.04
BMI (kg·m^−2^)	25	4		25	4		.63
IBW							
50–64 kg			11 (38)			13 (45)	.41
65–74 kg			11 (38)			8 (36)	
>75 kg			7 (24)			3 (19)	
ASA score							
1			2 (7)			3 (13)	.74
2			18 (62)			13 (54)	
3			9 (31)			8 (33)	
Analgesia before UG-FICB (n)			19 (66)			17 (71)	.68
Opiate (n)			17 (59)			15 (63)	
NSAID (n)			1 (4)			1 (4)	
S-Ketamine (n)			3 (10)			8 (33)	
Baseline NRS	4.0	2.0		4.7	1.9		.23
Hip fracture type							.22
Intracapsular			12 (41)			14 (58)	
Extracapsular			17 (59)			10 (42)	
Duration until operation (hours) – median [IQR]	16	5–22		15	5–21		

Values are shown as mean ± SD unless indicated otherwise; categorical data are presented as n (%). Ideal-body-weight (IBW) categories correspond to the weight-based dosing bands used in the trial protocol. The 2 groups were similar with respect to age, sex distribution, body-mass index (BMI), American Society of Anesthesiologists (ASA) physical-status class, prior analgesic administration, baseline numerical rating scale (NRS) pain score, and hip-fracture type. Time from emergency-department admission to surgery is reported as median [IQR].

ASA = American Society of Anesthesiologists, BMI = body-mass index, IBW = ideal body weight, IQR = interquartile range, NRS = numerical rating scale, NSAID = non-steroidal anti-inflammatory drug, SD = standard deviation, UG-FICB = ultrasound-guided fascia iliaca compartment block.

**Figure 2.**
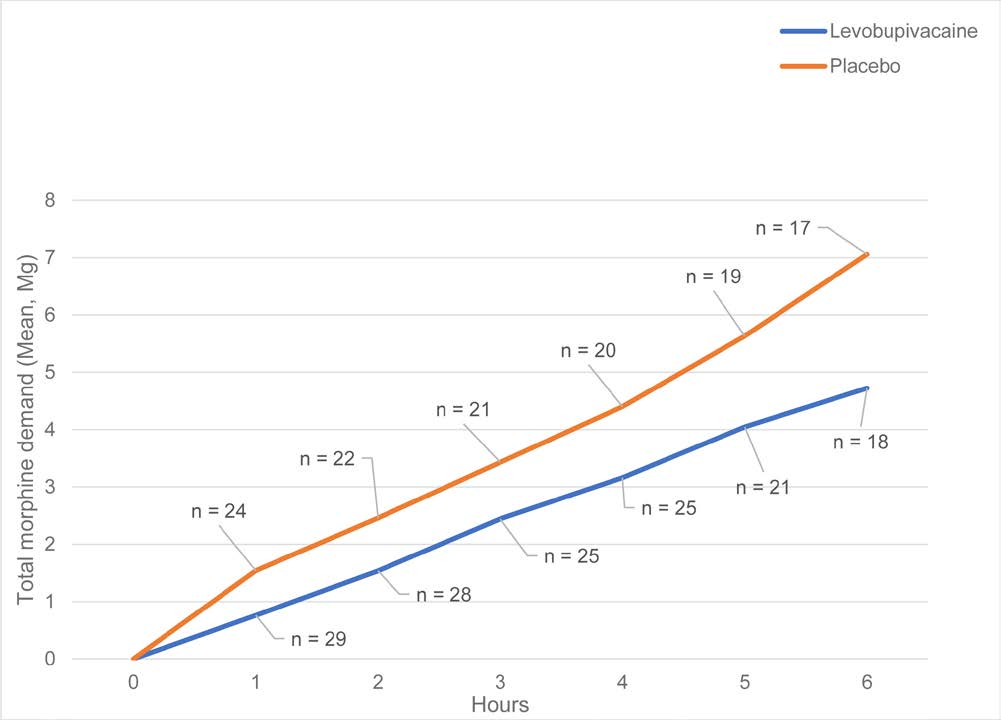
Cumulative intravenous-morphine use during the first six hours after emergency-department presentation for hip fracture. Solid red line = levobupivacaine (n = 29); solid blue line = placebo (n = 24). Patients were censored at hip surgery or at 6 h, whichever occurred first. A linear mixed-effects model showed no significant group difference (fixed-effect estimate –0.36 mg, *P* = .22).

Regarding the primary outcome, the cumulative morphine demand exhibited a median of 3 mg (IQR 1–6 mg) in the levobupivacaine group, versus 3 mg (IQR 2–6 mg) in the placebo group (*P* = .46). Mean cumulative morphine demand for the initial 6 hours is displayed in Figure 2. The linear mixed model analysis displayed a fixed effect estimate of −0.36 on morphine demand for the UG-FICB, which did not appear significant (*P* = .22).

**Figure 3.**
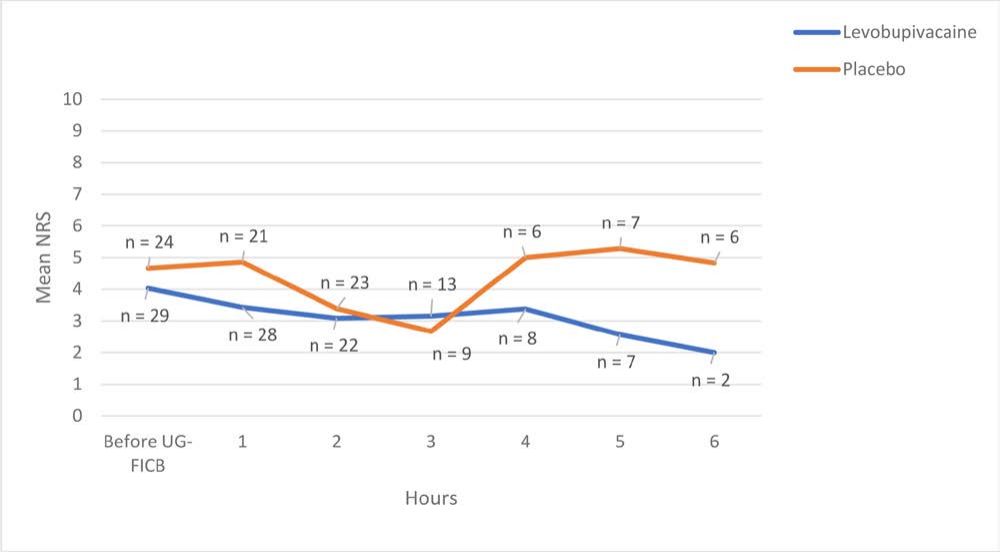
Pain on the numerical rating scale (NRS) during the first six hours after emergency-department presentation for hip fracture. Solid red line = levobupivacaine (n = 30); solid blue line = placebo (n = 25). Patients were censored at hip surgery or at 6 h, whichever occurred first. A linear mixed-effects model showed that pain was, on average, 0.91 NRS units lower with levobupivacaine than with placebo (*P* = .07). NRS = numerical rating scale.

During the first hour, participants from levobupivacaine group had an average NRS of 3.4 (SD 1.8) in contrast to the placebo group’s average NRS of 4.9 (SD 2.4) (*P* = .02). Figure 3 illustrates NRS scores for the initial 6 hours. The linear mixed model analysis for pain resulted in an estimate of fixed effect of −0.91 (*P* = .07) for the first 6 hours.

Of all participants, a notably higher proportion in the intervention group refrained from using additional morphine (24% vs 4%, *P* = .04; Table 4). Among participants who did use additional morphine, the median time until first dose was 43 minutes (IQR 15–122) in the intervention group versus 30 minutes (IQR 1–77) in the placebo group (*P* = .22).

**Table 4:** Analgesic efficacy and safety outcomes in the first 24 h after block placement.

	Levobupivacaine (n = 29)			Placebo (n = 24)			*P*
			n (%)			n (%)	
No morphine request			7 (24)			1 (4)	.04
Time until first morphine request (min) – median [IQR]	43	15–122	22	30	2–77	23	.21
Trial time (h) – mean (SD)	12	8		13	8		.72
NRS ≤ 3							
Until 6 h			12 (41)			5 (21)	.11
Until 24 h			7 (24)			4 (17)	.50
Adverse events (total)			2 (7)			4 (16)	.11
Pain with injection			0 (0)			1 (4)	
Nausea			0 (0)			1 (4)	
Vomiting			0 (0)			2 (8)	
Drowsiness			1 (3)			0 (0)	
Delirium			1 (3)			0 (0)	

Categorical outcomes are shown as n (%) and compared with Fisher exact test; continuous data are expressed as median [IQR] or mean ± SD and compared with the Mann–Whitney *U* test or Student *t* test, as appropriate.

Results. NRS = numerical rating scale. “No morphine request” indicates participants who remained opioid-free throughout follow-up. “Time until first morphine request” is reported in minutes among those who required rescue analgesia. “Trial time” reflects total observation time before surgery or 24 h, whichever occurred first. Adverse events were recorded up to surgery.

IQR = inter-quartile range, NRS = numerical rating scale.

**Figure 4.**
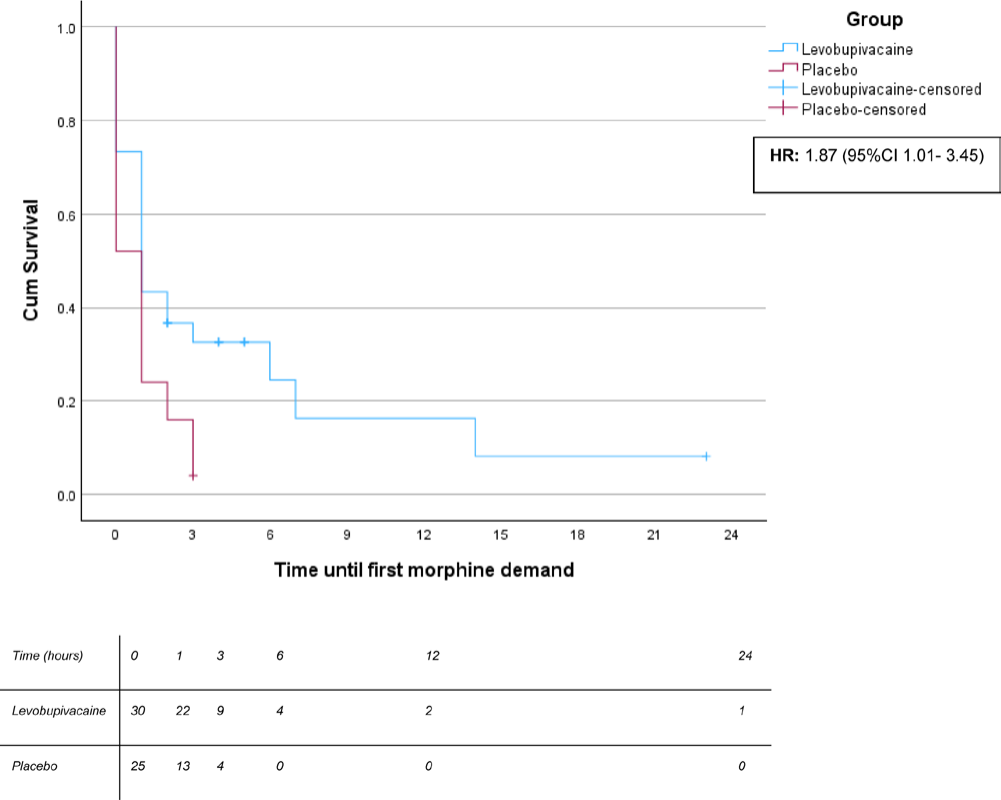
Results of time until first morphine demand after emergency-department presentation for hip fracture. Kaplan–Meier curves showing time to first morphine request in patients with hip fracture, comparing the levobupivacaine group (red line) and the control group (blue line). Time was measured from admission to either morphine request or censoring at the time of surgery or after 24 h without morphine with a log rank. Tick marks indicate censored observations. The number of patients at risk is shown below the x-axis at 0, 1, 3, 6, 12, and 24 h for each group. The hazard ratio for morphine request in the placebo group compared to levobupivacaine group was 1.87 (95% CI: 1.01–3.45), with a statistically significant difference between groups (*P* = .01, log-rank test).

These findings are consistent with the results of the Kaplan–Meier survival analysis (Fig. 4), which showed a statistically significant difference in time to first morphine request between groups (log-rank *P* = .01). Cox proportional hazards regression confirmed that patients in the placebo group had a significantly higher risk of morphine request compared to those in the levobupivacaine group (hazard ratio = 1.87, 95% CI: 1.01–3.45, *P* = .045).

Six participants experienced an adverse event, with 3 participants in the placebo group reporting nausea and vomiting. Another participant from this group encountered significant pain upon injection of the trial medication. The procedure was therefore halted when 15 mL was injected. Despite this, the participant completed the trial.

The last 2 participants, both from the intervention group, were unable to complete the trial due to their inability to manage the PCA-pump. One of them had developed delirium after 7 hours, while the other became drowsy after 6 hours. Recorded data up to the events were included in the analysis.

All participants who experienced an adverse event proceeded for hip surgery as planned and were included in all subsequent analyses.

Post hoc power calculations showed 17% power for the observed 6-hour morphine difference (*d* = 0.35). Including repeated measures in a mixed model increased estimated power to 49% (95% CI: 35–100).

## 4. Discussion

In this ED-based, double-blind, placebo-controlled RCT investigated whether UG-FICB with levobupivacaine reduces opioid demand in hip fracture patients.

The primary outcome, cumulative intravenous morphine demand via PCA during the first 6 hours, did not differ significantly between the levobupivacaine and placebo groups.

Despite this neutral finding, several secondary and exploratory analyses suggest a signal of analgesic effects. Survival analysis, for instance, showed that UG-FICB delayed the first request for morphine: the Kaplan–Meier curves diverged early (log-rank *P* = .01), and Cox modeling indicated almost a 2-fold higher hazard of morphine demand in the placebo group.

When looking at pain scores, there was a short-lived significant reduction in pain during the first hour and although the 6-hour linear mixed-model estimate narrowly missed significance, the direction of effect aligned with reduced pain.

### 4.1. Interpretation of findings

Several factors may explain why the apparent analgesic effect did not lead to a reduction in morphine demand. Pain was only NRS 4 at enrollment, and 68% of participants had already received systemic analgesia before the block. Earlier FICB trials report baseline scores of 6 to 8, even after pre-hospital analgesia, so our cohort began with markedly less residual pain, leaving little room for the block to demonstrate further analgesic effect.^[14]^

Finally, although every emergency physician completed training and at least ten supervised blocks, and published data show high success even with limited experience,^[8,26–28]^ a small study in ultrasound-guided regional anesthesia suggests that proficiency in needle visualization and block success is typically reached after 25 to 30 supervised procedures.^[29]^ As we did not archive ultrasound images or grade spread, partial or misplaced injections therefore remain a plausible source of dilution of effect.

Taken together, these factors could imply that the neutral primary result reflects contextual and methodological constraints – low baseline pain, pre-ED opioids, small sample size, and unverified block completeness – than an outright lack of efficacy of UG-FICB.

Our findings align with the 2 most recent reviews by Steenberg et al and Fadhlillah et al that found little analgesic benefit from FICB when pain at rest or nurse-administered morphine are the principle outcomes. In contrast, older landmark-guided studies, which often measured pain during leg movement, reported larger effects.^[14–18]^

Because most hip-fracture patients remain immobile before surgery, the clinical relevance of analgesia measured under forced mobilization is questionable.^[14–16]^ Our study therefore focused on opioid demand and pain at rest, outcomes that better mirror a real-world situation.

### 4.2. Limitations

Recruitment stopped early because of the COVID-19 pandemic, leaving the study markedly under powered: post hoc calculations indicated <20% power for the observed between-group difference in 6-hour morphine use and under 50% even when all repeated measures were modeled, so a type-II error cannot be ruled out.

Roughly one-third of patients proceeded to theater before the 6-hour time-point; although a mixed-effects model incorporated their data, residual bias may linger.

Missing pain scores – most often after evening admissions or in very elderly participants unfamiliar with the tablet interface – may also have diluted observable effects despite balanced group distribution. Finally, because the trial required comprehension of the numerical rating scale and operation of a PCA pump, patients with substantial cognitive impairment were not enrolled, limiting generalisability.

### 4.3. Future directions

Larger, fully powered trials, focusing on patients with higher baseline pain, are needed to clarify whether UG-FICB provides a clinically meaningful benefit in the emergency setting. Ideally continuous catheter-based FICB is used to cover the unpredictable interval to surgery.

## 5. Conclusion

In this under-powered RCT, a single-shot UG-FICB did not significantly lower 6-hour opioid demand or resting pain after hip fracture presentation, although it delayed the first morphine dose and allowed one in 4 patients to remain opioid free.

Larger, adequately powered trials, ideally exploring continuous catheter techniques and enrolling patients with higher baseline pain, are needed to support widespread adoption.

## Acknowledgments

We extend our gratitude to the following individuals for their invaluable contributions to this trial: E. Bergman PhD, D. Fransz, S MD PhD, E. van Gurp, P. Hustinx MD PhD, A. Merry PhD, R. J. Willems MD, B.C. van der Zwaard PhD, F. al Khoury MD, D. Bussmann-Willems MD, L. Claassen MD, L. Colen-Kroon MD, M. Franssen MD, M. Klein Ovink MD, G. Latten MD PhD, G. Raven MD, I. Verrijth-Wilms MD and A. van Tienen MD. We also express our appreciation to all the other staff members of the ED, wards, hospital pharmacy and trial center of the Zuyderland Medical Centre for their involvement. In conclusion, we would like to express our profound gratitude to the SGO Foundation and Fresenius Kabi Netherlands B.V. for their generous contributions, which made this study possible.

During revision the authors used ChatGPT (OpenAI, San Francisco, CA) as a writing aid. The model helped streamline wording and eliminate repetition, and propose clearer paragraph structure for different sections in the manuscript. All suggestions were critically reviewed, edited, and approved by the authors; no new data, analyses, or scientific conclusions were generated by the AI tool.

## Author contributions

**Conceptualization:** Rory D. O’Connor, Sanne Postma, Djamila van Kamp, Alicia Lommen, Jan A. Ten Bosch, Mattheus K. Reinders.

**Data curation:** Rory D. O’Connor, Djamila van Kamp, Annelies C.E. Benraad, Sarah Körver, Mattheus K. Reinders, Sanne Postma.

**Formal analysis:** Rory D. O’Connor, Alicia Lommen, Mattheus K. Reinders.

**Funding acquisition:** Rory D. O’Connor, Sanne Postma.

**Investigation:** Rory D. O’Connor, Annelies C.E. Benraad, Sarah Körver, Mattheus K. Reinders, Sanne Postma.

**Methodology:** Rory D. O’Connor, Sanne Postma, Djamila van Kamp, Annelies C.E. Benraad, Alicia Lommen, Sarah Körver, Mattheus K. Reinders.

**Project administration:** Rory D. O’Connor, Djamila van Kamp, Annelies C.E. Benraad, Alicia Lommen, Mattheus K. Reinders.

**Resources:** Rory D. O’Connor, Sanne Postma, Djamila van Kamp, Mattheus K. Reinders.

**Software:** Rory D. O’Connor.

**Supervision:** Rory D. O’Connor, Sanne Postma, Sarah Körver, Jan A. Ten Bosch, Mattheus K. Reinders.

**Validation:** Rory D. O’Connor, Sanne Postma, Djamila van Kamp, Annelies C.E. Benraad, Alicia Lommen, Sarah Körver.

**Visualization:** Rory D. O’Connor.

**Writing – original draft:** Rory D. O’Connor, Mattheus K. Reinders, Sanne Postma.

**Writing – review & editing:** Rory D. O’Connor, Sanne Postma, Djamila van Kamp, Annelies C.E. Benraad, Alicia Lommen, Sarah Körver, Jan A. Ten Bosch, Mattheus K. Reinders.
